# Encountering sexual and gender minority youth in healthcare: an integrative review

**DOI:** 10.1017/S146342361900001X

**Published:** 2019-03-20

**Authors:** Minna Laiti, Anni Pakarinen, Heidi Parisod, Sanna Salanterä, Salla Sariola

**Affiliations:** 1 Department of Nursing Science, Faculty of Medicine, 20014 University of Turku, Turku, Finland; 2 The Nursing Research Foundation (NRF), Asemamiehenkatu 2, Helsinki, Finland; 3 Turku University Hospital, Turku, Finland; 4 Faculty of Political sciences, Discipline of Sociology, 00014 University of Helsinki, Helsinki, Finland

**Keywords:** adolescent, adolescent health services, review, sexual and gender minorities

## Abstract

**Aim:**

To describe the encounters with sexual and gender minority (SGM) youth in healthcare based on the existing research.

**Background:**

The development of sexual orientation and gender identity can create challenges in an SGM youth’s life, and they may need support from health professionals. Heteronormativity has been recognised as a barrier to the identification of diversity in sexuality and gender, and no previous literature review has studied heteronormativity thoroughly.

**Methods:**

An integrative review following Whittemore and Knafl was conducted. A literature search was systematically undertaken in six databases (PubMed/MEDLINE, CINAHL, Cochrane Library, PsycINFO, Eric, and Academic Search Premier). Finally, 18 research articles were included. Data were analysed deductively with the theoretical framework from Stevi Jackson’s (2006) article to understand the role of heteronormativity in the healthcare of SGM youth.

**Findings:**

The encounters with SGM youth consisted of two simultaneous themes. *Heteronormative care* included three elements: (1) the effect of heteronormativity on health professionals’ competence to work with SGM youth, (2) false assumptions about SGM youth, and (3) the influence of heteronormativity on encounters with SGM youth. *Diversity-affirming care* included two elements: (4) the considerateness of health professionals towards SGM youth and (5) inclusive care of SGM youth.

**Conclusion:**

This review summarised how SGM youth were encountered in healthcare and how heteronormativity was affecting their healthcare. Furthermore, this review identified elements that supported diversity-affirming care. With diversity-affirming care, SGM youth may access the information and support they need from healthcare. Further research is needed about how diversity-affirming care can be applied to the healthcare of SGM youth and how elements of heteronormative care are occurring globally in the healthcare of SGM youth. The perceptions of transgender and other gender minority youth were under-represented in the studies and research needs to focus more on how they are encountered in healthcare.

## Introduction

During adolescence, a period between the ages of 10 and 19 years, young people go through several physical, psychosocial, and sexual maturation changes, including the development of gender identity and sexual orientation (World Health Organization, 2017; World Health Organization, 2018a). Here, we use the term sexual and gender minority (SGM) youth to refer to adolescents whose sexual orientation is non-heterosexual and/or their gender identity is outside the female/male binary (Smalley *et al*., 2018). In adolescence, SGM youth may face challenges that are related to their identity (The Lancet, 2011; Bregman *et al*., 2013). They may face victimisation and bullying (Espelage *et al*. 2008; Huebner *et al*., 2015; Kosciw *et al*., 2015), as well as negative attitudes from peers and family (Bregman *et al*., 2013; Kosciw *et al*., 2015; Katz-wise *et al*., 2016; Puckett *et al*., 2017). To be able to face these challenges, SGM youth may benefit from the support of various professionals including health professionals (Reynolds, 2011). Appropriate support requires, for example, professionals’ competency, respectful attitude, and ability to fulfil SGM youths’ rights to information, privacy, confidentiality, and non-discrimination in healthcare (World Health Organization, 2015).

In healthcare, SGM people are often an invisible patient group (Fish and Bewley, 2010; McIntyre and McDonald, 2012). One reason for this is heteronormativity, defined as a general assumption that everyone is heterosexual and everything else is exceptional (Fish and Bewley, 2010; McIntyre and McDonald, 2012; Katz, 2014). Norms, including heteronormative ones, are factors that are generally thought to guide human actions (values, beliefs, assumptions). They operate within social dimensions such as cultural, institutional, sexual, and/or interpersonal. Heteronormativity defines normative ways of life as well as normative ways of sexuality. It can be examined from three aspects: gender, sexuality, and heterosexualness (Jackson, 2006). First, gender is a state of being female/male in social and cultural levels, and sex refers to biological levels of being female/male (Smalley *et al*., 2018). In heteronormativity, gender is usually seen through a binary, where man and woman are opposites completing each other. Gender includes acts that are assumed to cohere with biological sex normatively, such as women being feminine and men being masculine. Second, sexuality is supposed to cohere with gender, as in women and men sexually desiring each other as complementary genders. Finally, heterosexualness is both sexual acts that are ‘natural and normal’ and non-sexual acts, such as a heterosexual woman/man having a biological need to reproduce (Jackson, 2006). Thus, young people who have a sexual orientation and/or gender identity that cannot be understood through heteronormativity, may experience invisibility in different fields of society, including healthcare. There are no earlier literature reviews that have summarised the encounters with SGM youth in healthcare. Thus, *the aim of this study was to describe the encounters with SGM youth in healthcare based on the existing research*. Furthermore, this study focuses for the first time on the role of heteronormativity and its relation to encountering SGM youth in healthcare.

## Methods

An integrative review is a well-suited method for combining research with diverse methods (Whittemore and Knafl, 2005). It can give a new, comprehensive understanding of a specific phenomenon (Torraco, 2005; Whittemore and Knafl, 2005). An integrative review was conducted following Whittemore and Knafl’s (2005) five-stage process: (1) problem identification, (2) literature search, (3) data evaluation, (4) data analysis, and (5) presentation of results (Whittemore and Knafl, 2005).

### Problem identification

In the problem identification stage, a clear aim for the review was defined to support the following stages (Whittemore and Knafl, 2005). The aim was to describe the encounters with SGM youth in healthcare based on existing research, and, to make a comprehensive review, no time limit regarding publication date was set.

### Literature search

The literature search stage includes precise planning and description of the search strategy a priori to maintain the rigour in the review (Torraco, 2005; Whittemore and Knafl, 2005). A systematic search was undertaken between the 24^th^ of November, 2017, and the 1^st^ of January, 2018, in six electronic databases: PubMed/MEDLINE, CINAHL, Cochrane Library, PsycINFO, Eric, and Academic Search Premier. Search phrases included combinations of key terms and free-text terms for the following concepts: *sexual and gender minorities* (e.g. homosexual, transgender persons), *adolescents* (e.g. youth, teen), and *healthcare practices* (e.g. school healthcare, adolescent health services). The detailed search strategy is in Table 1.

**Table 1:** Search phrases in the literature search

Database	Search phrase
**PubMed/Medline**	(“School Health Services”[Mesh] OR “school health” OR “school healthcare” OR “school nurs*“ OR “Nursing Care”[Mesh] OR “Nursing”[Mesh] OR “nursing” OR “Adolescent Health Services”[Mesh] OR nurse* OR “healthcare”) AND (“Adolescent”[Mesh] OR adolescen* OR youth OR teen* OR young) AND (“Sexual Minorities”[Mesh] OR “Transgender Persons”[Mesh] OR homosex* OR lesb* OR bisex* OR transsex* OR transgender* OR queer OR intersex* OR asex*)
**CINAHL**	AB ((MH “School Health Services+“) OR (MH “Nursing Care+“) OR (MH “Adolescent Health Services”) OR (MH “School Health Nursing”) OR “school health” OR “school healthcare” OR “school nurs*“ OR “nursing” OR nurse* OR “healthcare”) AND ((MH “Adolescence+“) OR (MH “Young Adult”) OR adolescen* OR youth OR teen* OR young) AND ((MH “Transgender Persons+“) OR (MH “GLBT Persons+“) OR (MH “Gay Persons+“) OR (MH “Homosexuality”) OR (MH “Gay Men”) OR (MH “Lesbians”) OR (MH “Bisexuality”) OR homosex* OR lesb* OR bisex* OR transsex* OR transgender* OR queer OR intersex* OR asex*))
**Cochrane Library**	([mh “School Health Services”] OR [mh “Nursing Care”] OR [mh “Adolescent Health Services”] OR “school healthcare” OR “school nurse” OR nursing OR healthcare) AND ([mh Adolescent] OR adolescent OR youth OR teenager OR young) AND ([mh “Sexual and Gender Minorities”] OR [mh “Transgender Persons”] OR [mh Homosexuality] OR [mh Bisexuality] OR homosexual OR lesbian OR bisexual OR transsexual OR transgender OR queer OR intersex OR asexual)
**PsycINFO**	exp Nurses/ or exp Nursing/ or exp Health Care Services/ or school health*.mp. or nursing.mp. or school nurs*.mp. or nurse*.mp. or healthcare.mp. and (adolescen* or youth or teen* or young).mp. and exp Sexual Orientation/ or exp Bisexuality/ or exp Homosexuality/ or exp Gender Identity/ or exp Lesbianism/ or exp Transsexualism/ or exp Male Homosexuality/ or exp Transgender/ or homosex*.mp. or lesb*.mp. or bisex*.mp. or transsex*.mp. or transgender*.mp. or queer.mp. or intersex*.mp. or asex*.mp.
**Eric**	(DE “School Health Services” OR DE “Nursing” OR DE “Community Health Services” OR DE “School Health Services” OR DE “Health Services” OR DE “Medical Services” OR DE “School Nurses” OR DE “Nurses” OR “school health” OR “school healthcare” OR “school nurs*“ OR “nursing” OR nurse* OR healthcare) AND (DE “Adolescents” OR adolescen* OR youth OR teen* OR young) AND (DE “Sexual Orientation” OR DE “Sexual Identity” OR DE “Homosexuality” OR homosex* OR lesb* OR bisex* OR transsex* OR transgender* OR queer OR intersex* OR asex*)
**Academic Search Premier (EBSCO)**	(DE “MEDICAL care” OR DE “CHILD health services” DE “COMMUNITY health services” OR DE “EMERGENCY medical services” OR DE “HOSPITAL care” OR DE “PRIMARY health care” OR DE “SCHOOL health services” OR DE “STUDENT health services” OR DE “NURSING services” OR “school health” OR “school healthcare” OR “school nurs*“ OR “nursing” OR nurse* OR healthcare) AND (DE “TEENAGERS” OR DE “ADOLESCENCE” OR DE “YOUTH” OR adolescent OR youth* OR teen* OR young) AND (DE “LGBT high school students” OR DE “BISEXUAL high school students” OR DE “GAY high school students” OR DE “TRANSGENDER high school students” OR DE “LGBT youth” OR DE “BISEXUAL youth” OR DE “GAY youth” OR DE “LESBIAN youth” OR DE “LGBT teenagers” OR DE “LGBT young adults” OR DE “TRANSGENDER youth” OR DE “LGBT people” OR DE “BISEXUALS” OR DE “GAY people” OR DE “LESBIANS” OR DE “LGBT students” OR DE “LGBT youth” OR DE “MINORITY LGBT people” OR DE “TRANSGENDER people” OR homosex* OR lesb* OR bisex* OR transsex* OR transgender* OR queer OR intersex* OR asex*)

The inclusion criteria for the literature were as follows: (1) *a scientific, peer-reviewed research article*, (2) *the publication language was English*, (3) *a focus on the perspectives of SGM youth in healthcare practices,* or (4) *a focus on the perspectives of health professionals working with SGM youth*. In this review, health professionals were defined as graduated professionals who are working in the field of healthcare. The article was excluded if (1) *more than 50% of SGM youth participants were younger than 10 years or older than 19 years*, (2) *the study focussed on a medical condition (*e.g. *HIV)*, or (3) *the study focussed on a health problem (*e.g. *tobacco or substance use)*. The latter two exclusion criteria based on the perception that studies focussing solely on a medical condition or a health problem do not include the perspectives of SGM youth or health professionals.

**Figure 1:**
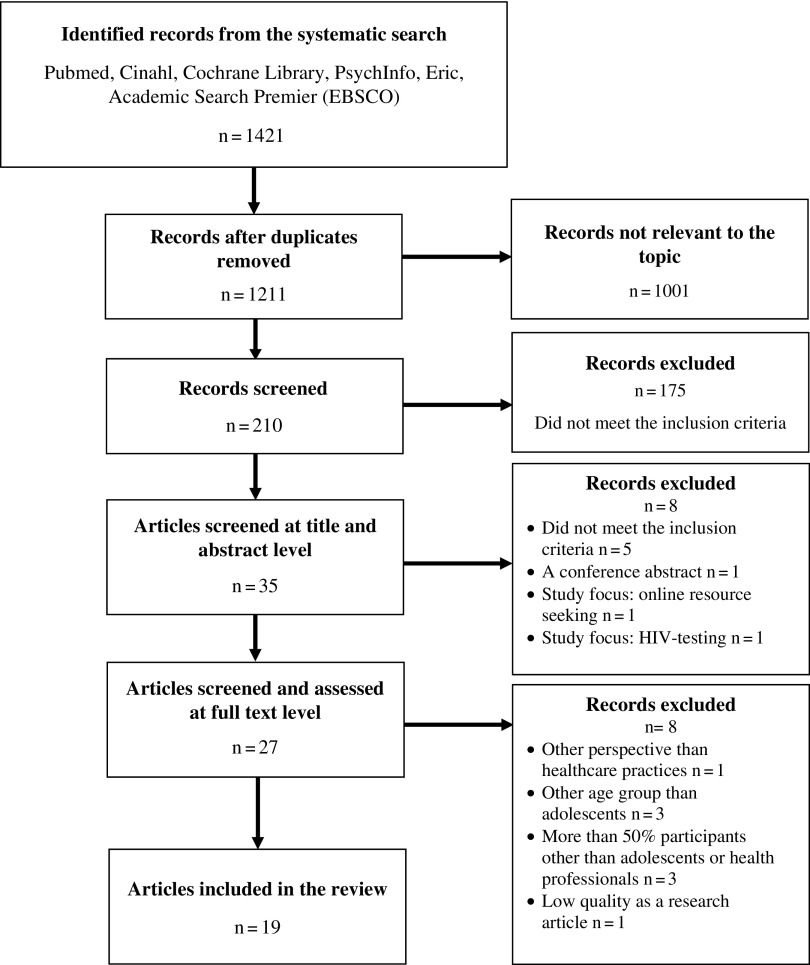
Flow diagram of the article screening and selection

The total results of the systematic search included 1421 scientific, peer-reviewed research articles that were published in 1978–2017. Screening of the articles was done first at the title and the abstract level, and second at the full text level (Figure 1). Two authors screened the articles independently. During the screening process, the authors discussed the eligibility of the remaining articles. Some articles were excluded because the abstract was not available, or an exclusion criterion was fulfilled. One article was considered as ‘a borderline case’ because it was uncertain if 50% of the participants were 10–19 years old due to age group categories. However, because 40% of the participants were certainly 10–19 years old and the article was congruent with other inclusion criteria and the aim of the study, the article was included. Finally, 18 scientific research articles were included in the analysis.

### Data evaluation

The chosen articles were extracted, and the quality of studies was appraised (Whittemore and Knafl, 2005). The articles were extracted into two tables with key information about the studies. Table 2 describes studies conducted from SGM youth’s perspectives, and Table 3 includes studies conducted from health professionals’ perspectives (Tables 2 and 3).

**Table 2:** The studies viewing healthcare from the perspective of SGM youth

Reference	Country	Study aim	Design	Study sample	Method/s	Key findings	Quality score with MMAT (%)
Scherzer (2000)	United States	Examine young lesbian and bisexual women’s constructions of health and their lived experiences focused on the medical healthcare interactions	A qualitative descriptive study	Lesbian and bisexual women aged 18–21. Sample size *n*=8	Semi-structured interviews	Three themes related to barriers to lesbian and bisexual women seeking medical care were highlighted: (1) the agency exercised by young lesbian and bisexual women, (2) the impact of healthcare providers on women’s access to and utilisation of medical care, and (3) reflections of healthcare interactions to larger power dynamics in society	100
Ginsburg *et al*. (2002)	United States	Examine factors that make sexual minority youth feel safe in healthcare settings	A mixed-methods study	LGBTQ youth aged 14–23 years. Sample sizes in four-stage research process; in Stage 1 *n*=8, Stage 2 *n*=72, Stage 3 *n*=94, and in Stage 4 *n*=41	Stage 1 Expert focus groups, Stage 2 Nominal group technique, Stage 3 Survey, and Stage 4 Open focus groups	Most of important factors for LGBTQ youth were the same as for other youth, and factors were related to healthcare professional’s characteristics, and professionals should meet youth open-mindedly. Professionals should also have more knowledge about sexual minority youth	25
Hoffman *et al*., (2009)	United States and Canada	Determine preferences of LGBTQ youth regarding healthcare providers, healthcare settings and health issues which are important for youth to discuss with a healthcare provider	A quantitative descriptive study	LGBTQ youth aged 13–21 years. Sample size *n*=733	A cross-sectional web-based questionnaire	Most important quality in a healthcare provider was interpersonal skills. Most important things in healthcare settings were general things such as cleanliness. Mental health, physical health and STD issues were the most important topics to discuss with a provider	75
Rasberry *et al*., (2015)	United States	Help inform school-centred strategies for connecting Black and Latino young men who have sex with men (YMSM) to HIV and STD prevention services. This was provided by describing (1) the willingness and safety of YMSM to discuss sexual health and sexual orientation-related topics, (2) the experiences of YMSM with school nurses discussing about sexual health-related topics	A mixed-methods study	Black and Latino YMSM aged 13–19 years. Sample size *n*=447	A web-based questionnaire, and in-depth, semi-structured interviews that covered same topics as the questionnaire	YMSM were willing to talk school staff about sexual health topics. However, they were not willing to talk if staff’s opinions about sexual minorities were uncertain, or they lacked knowledge in LGBTQ issues. YMSM felt least safe to talk about their sexual orientation to school nurses. The school nurse was often described as a limited care provider, a rare visitor in the school, and whose personality did not seem to be open and caring	75
Arbeit *et al*. (2016)	United Sates	Analyse bisexual female youth’s experiences accessing sexual health information and services provided by a doctor, nurse, or a counsellor	A mixed-methods study	Cisgender, bisexual female youth aged 14–17 years. Sample size *n*=40	An online questionnaire and asynchronous online focus groups	Aspects in provider’s behaviour affecting mostly participants’ experiences were: (1) negative bias to adolescent sexual behaviour and same-sex attraction, (2) heterosexual assumptions about youth, (3) missed opportunities to screen for HIV and STIs. Bisexual stigma within families was associated with the disclosure to a provider. School-based sexual health information was limited on abstinence and condoms	25
Fuzzell *et al*. (2016)	United Sates	Examine sexual minority and majority youth and young adult’s experiences of communication with a physician about sexuality, and what advice youth give for improving interactions	A qualitative descriptive study	Sexual minority and majority youth and young adults aged 12–31. Sample size *n*=40	Semi-structured interviews	Five main themes arose from the interviews: (1) need for increased quantity of sexual communication, (2) need for increased quality of sexual communication, (3) concerns about confidentiality/privacy, (4) comfort, and (5) inclusivity	75
Snyder *et al*. (2016)	Canada	Determine LGBTQ youth’s experiences with primary care physicians, identify gaps in primary healthcare services and areas for improvement	A mixed-methods study	LGBTQ youth aged 14–18+ years. Sample size *n*=60	A paper-pencil survey and focus-groups discussion	LGBTQ youth’s healthcare needs were not met well, and they had experiences of poor patient-provider communication, disrespect, and lack of discussions about important topics such emotional and sexual health. Concern of confidentiality and inappropriate comments were identified as barriers to care. The same items stated above were also mentioned as areas for improvement	50
Rose and Friedman (2017)	United States	Examine African American sexual and gender minority youth males’ perceptions about school sexual health education and services	A qualitative descriptive study	African American sexual and gender minority youth aged 18–21 years. Sample size *n*=42	Semi-structured focus groups and in-depth interviews	Participants indicated that schools have missed opportunities to educate sexual and gender minority youth about sexual health including sexual orientation. Participants had controversial perceptions about school-based health services, only limited information was offered to them, and school nurses did not always have knowledge about health issues that impact this youth	50

**Table 3:** The studies examining health professionals working with SGM youth

Reference	Country	Study aim	Design	Study sample	Method/s	Key findings	Quality score with MMAT (%)
East and El Rayess (1998)	United States	Investigate the physician-patient relationship between paediatricians and lesbian, gay or bisexual youth	A quantitative descriptive study	Paediatricians Sample size *n*=60	A paper-pencil questionnaire	Most paediatricians had reservations about approaching the issue of sexual orientation, such as it may offend the patient, or they do not know enough about the needs of lesbian, gay, and bisexual youth. Most paediatricians reported they would not break patient confidentiality to inform parents about patient’s same-sex sexual activity unless under extreme circumstances. Paediatricians need and desire for further training about the healthcare of these youth	75
Sawyer *et al*. (2006)	United States	Conduct a national-level needs assessment for developing training programs and educational materials for school health professionals to better meet the needs of GLBQ students	A quantitative descriptive study	School counsellors, school nurses, school psychologists, and social workers Sample size *n*=941	A paper- pencil survey	Most health professionals indicated that GLBQ students exist in their schools. Professionals’ attitudes towards GLBQ students were accepting and tolerant. Professionals indicated that GLBQ students have a greater risk for certain health problems such as mental health issues. Results show that health professionals see their role important in providing health and mental health services for GLBQ students, but they indicate they lack of knowledge, training, and skills to work with GLBQ students	50
Kitts, (2010)	United States	Identify barriers to optimal care between physicians and LGBTQ youth	A quantitative correlational study	Physicians in paediatrics, internal medicine, obstetrics-gynecology, psychiatry, emergency medicine, and family practice Sample size *n*=184	A paper-pencil survey	The majority of physicians would not discuss regularly about youth’s sexual orientation, sexual attraction, or gender identity when they took a sexual history from a sexually-active adolescent. Some physicians were not aware of an association between LGBTQ youth and suicide. The majority of physicians indicated a lack of skills to address issues of sexual orientation with adolescents, and they agreed that sexual orientation needs to be addressed more often with adolescents	25
Rose and Friedman (2013)	United States	Review systematically literature which has focused on the health information seeking practices by sexual minority youth	A qualitative descriptive study	Empirical studies from medical/health and sociology databases. Sample size *n*=19	A systematic review	Most commonly used source of health information was healthcare providers. However, several studies described the limited availability of targeted health information developed for sexual minority youth. The health needs of sexual minority youth were ignored due to lack of knowledge about health issues specific to them	75
Knight *et al*. (2014)	Canada	Examine clinicians’ experiences providing sexual health services for LGBTQ youth	A qualitative descriptive study	Clinicians (doctors and nurses) from clinics that specialised in providing sexual health services for youth Sample size *n*=24 (5 doctors and 19 nurses)	In-depth, semi-structured interviews	Clinicians indicated that they were not adequately equipped to provide culturally competent sexual health services for LGBTQ youth. Clinicians mentioned reasons for this situation: a lack of knowledge about LGBTQ youth, a lack of institutional support and resources even though clinicians were willing to develop programs for responding the needs of LGBTQ youth	75
Mahdi *et al*. (2014)	United States	Describe school health professionals’ preparedness to address needs of LGBTQ students. Preparedness consisted of knowledge, attitudes and skills	A quantitative correlational study	School nurses, counsellors and social workers who attended in New Mexico school health conference Sample size *n*=183	A self-administered survey and The Attitudes Toward Lesbian and Gay Men (ATLG) scale	The majority of professionals had moderate or high knowledge of LGBTQ youth behavioural health risks. The majority of professionals had more confidence to work with LGBTQ than experience about discussing behavioural health concerns with LGBTQ youth.School nurses had the lowest knowledge of LGBTQ students at being risk for suicide, depression, and discrimination. School counsellors and social workers’ lowest knowledge was about LGBTQ community-based organisations, or counsellors experienced with LGBTQ concerns The majority had positive attitudes toward gay and lesbians. School nurses were more likely to have negative attitudes toward gays and lesbians when compared with other professionals’ attitudes	50
Torres *et al*. (2015)	United States	Understand better providers’ perspectives in healthcare needs and barriers to care for transgender youth	A qualitative descriptive study	Providers (clinical staff, researchers and trained community educators) who interact closely with transgender youth Sample size *n*=11	In-depth interviews	Providers of transgender youth recognised multiple barriers and challenges in the care of this youth: lack of access to services, the critical role of support, challenges in navigating in the healthcare system, and the need for training about trans-affirming care. However, the providers identified the resilience of transgender youth despite the obstacles they have faced	75
Vance *et al*. (2015)	United States	Explore healthcare providers’ clinical experiences, comfort with, confidence and barriers in providing care to transgender youth	A quantitative descriptive study	Healthcare providers, who were members of the Society for Adolescent Health and Medicine and the Pediatric Endocrine Society Sample size *n*=475	An online survey	Over half of providers had provided care to transgender youth and felt comfortable with providing medical care to this youth, but under half of providers felt confident in doing so. Barriers in providing transgender-related care were a lack of several things such as training, and exposure to transgender patients	25
Lefkowitz and Mannell (2017)	United Kingdom	Examine sexual health providers’ perceptions of transgender youth as social presentations and how these presentations could impact the treatment of transgender youth, their health and well-being	A qualitative descriptive study	Service providers Sample size *n*=20	Semi-structured interviews	Providers understood transgender through male/female binary, and linked transgender identity with homosexuality, and providers had conflicting perceptions about transgender bodies and sexual organs. Many providers thought transgender youth were mentally unstable such as distressed or depressed and they face difficulties in society’s acceptance. Many providers indicated also that transgender youth were too young to be certain their gender identity	100
Shires *et al*. (2017)	United States	Explore general paediatricians’ experience with and willingness to manage transgender youth’s gender-affirming care, which included puberty blockers and cross-sex hormones	A quantitative descriptive study	General paediatricians Sample size *n*=21	An online survey	Approximately half of the paediatricians had provided care for an adolescent with gender dysphoria. None of the paediatricians had prescribed puberty blockers or initiated cross-sex hormone therapy, and only one-third were willing to learn about these medications More than half of paediatricians would refer transgender youth to another provider regarding puberty blockers and hormone therapy. Barriers to caring transgender youth included a lack of time, a lack of training in transgender-specific care, and a lack of familiarity with guidelines for gender-affirming care	25

To appraise the quality of the studies, the Mixed Methods Appraisal Checklist (MMAT) tool was used. Pluye *et al*. (2011) designed the MMAT tool to appraise the quality of qualitative, quantitative, and mixed methods studies in literature reviews (Pluye *et al*., 2011; Souto *et al*., 2015). A mixed methods study is a study that uses both qualitative and quantitative data collection methods, based on the definition in the MMAT tool. Quality appraisal started with evaluating criteria that were common to all studies (research questions) and specific to research design (data collection and findings). For each criterion, response options were ‘yes’, ‘no’, or ‘can’t tell’. Next, to score the quality, all ‘yes’ answers were summed, and then divided by the total number of criteria. Finally, the number was multiplied by 100, giving the total percentage score of quality. Two authors (ML, AP) did the quality appraisal independently and, through discussion, achieved consensus about the quality. These final scores were summed into Tables 2 and 3.

### Data analysis

A deductive descriptive data analysis was performed (Whittemore and Knafl, 2005; Whittemore, 2007). Jackson’s (2006) theoretical article was used as a theoretical framework to understand the role of heteronormativity in the healthcare of SGM youth. Based on this understanding, the elements addressing heteronormativity and breaking heteronormativity were identified from the studies, listed, and compared together. Finally, two themes with five elements related to heteronormativity and the encounters with SGM youth were identified.

## Results

### Study characteristics

The studies used a variety of research methods: qualitative interview (Scherzer, 2000; Knight *et al*., 2014; Torres *et al*., 2015; Fuzzell *et al*., 2016; Lefkowitz and Mannell, 2017; Rose and Friedman, 2017) and qualitative questionnaire (East and El Rayess, 1998) studies, mixed methods studies (Ginsburg *et al*., 2002; Rasberry *et al*., 2015; Arbeit *et al*., 2016; Snyder *et al*., 2016), quantitative survey studies (Sawyer *et al*., 2006; Hoffman *et al*., 2009; Kitts, 2010; Mahdi *et al*., 2014; Vance *et al*, 2015; Shires *et al*., 2017), and a literature review (Rose and Friedman, 2013). More than half of the studies were done from the perspective of health professionals (East and El Rayess, 1998; Sawyer *et al*., 2006; Kitts, 2010; Rose and Friedman, 2013; Knight *et al*., 2014; Mahdi *et al*., 2014; Torres *et al*., 2015; Vance *et al*., 2015; Lefkowitz and Mannell, 2017; Shires *et al*., 2017), four of which focussed on the healthcare of transgender youth (Torres *et al*., 2015; Vance *et al*, 2015; Lefkowitz and Mannell, 2017; Shires *et al*., 2017). One study was conducted before the 2000s (East and El Rayess, 1998). Studies were done in the United States (East and El Rayess, 1998; Scherzer, 2000; Ginsburg *et al*., 2002; Sawyer *et al*., 2006; Hoffman *et al*., 2009; Kitts, 2010; Rose and Friedman, 2013; Mahdi *et al*., 2014; Rasberry *et al*., 2015; Torres *et al*., 2015; Vance *et al*., 2015; Arbeit *et al*., 2016; Fuzzell *et al*., 2016; Rose and Friedman, 2017; Shires *et al*., 2017), in Canada (Hoffman *et al*., 2009; Knight *et al*., 2014; Snyder *et al*., 2016), and in the United Kingdom (Lefkowitz and Mannell, 2017).

Most studies considered SGM youth as *a uniform adolescent minority group*, and defined them as *lesbian, gay, bisexual, transgender, and questioning* (LGBTQ) (Ginsburg *et al*., 2002; Sawyer *et al*., 2006; Hoffman *et al*., 2009; Kitts, 2010; Mahdi *et al*., 2014; Fuzzell *et al*., 2016; Snyder *et al*., 2016), or *queer* (Scherzer, 2000; Knight *et al*., 2014; Snyder *et al*., 2016). Several studies identified diversity in sexuality and sexual orientation; the youth were able to describe their identity through attraction, identity, experience, or behaviour (Arbeit *et al*., 2016; Snyder *et al*., 2016) and could specifically describe their identity with the terms *dyke*, *pansexual* (Scherzer, 2000; Fuzzell *et al*., 2016; Snyder *et al*., 2016), or just *other* (Ginsburg *et al*., 2002; Fuzzell *et al*., 2016). Some studies did not categorise identities at all (Rasberry *et al*., 2015; Rose and Friedman, 2017). East and El Rayess (1998) and Rose and Friedman (2013) focussed solely on sexual minority youth which they defined as gay, lesbian, and bisexual youth. Ginsburg *et al*. (2002) and Snyder *et al*. (2016) defined transgender as a sexual orientation. Related to gender identity, the term *cisgender* was identified in the studies of Arbeit *et al*. (2016) and Shires *et al*. (2017).

### Quality assessment

The quality scores ranged from 25% to 100% according to the MMAT tool. This range indicated that the quality of the studies was low to excellent. The most frequent score was 75% (*n*=7), and the quality of Scherzer’s (2000) and Lefkowitz and Mannell (2017) studies was assessed as excellent with a score of 100%. The quality appraisal did not exclude any articles, but it gave an overview of the quality in studies in contrast with the results (Pluye *et al*., 2011).

### The themes of encountering SGM youth in healthcare

**Figure 2:**
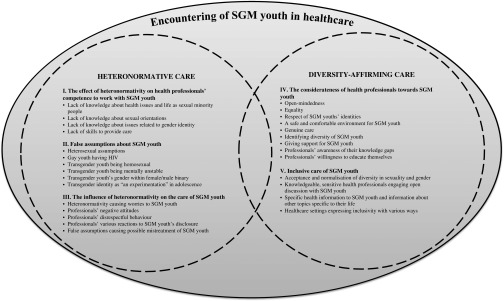
The themes of encountering SGM youth in healthcare

Based on the data analysis, the encounters with SGM youth in healthcare consisted of two themes ***heteronormative care*** and ***diversity-affirming care***. This shows how the healthcare of SGM youth does not only include either heteronormative or heteronormativity-breaking elements. Both heteronormative care and diversity-affirming care have their own specific elements. These five elements include (1) *the effect of heteronormativity on health professionals’ competence to work with SGM youth*, (2) *false assumptions about SGM youth*, (3) *the influence of heteronormativity on encounters with SGM youth,* (4) *the considerateness of health professionals towards SGM youth,* and (5) *inclusive care of SGM youth*. The first three elements describe the heteronormative care, and the last two describe the diversity-affirming care. The encounters with SGM youth in healthcare can include elements from both themes (Figure 2).

### Heteronormative care

#### Element 1. The effect of heteronormativity on health professionals’ competence to work with SGM youth

Most studies identified problems in health professionals’ competence to work with SGM youth. These problems can be related to heteronormativity. Scherzer (2000), Rasberry *et al*. (2015), and Rose and Friedman (2017) reported that SGM youth experienced health professionals lacking knowledge about SGM-relevant health issues and living as sexual minority people. Fuzzell *et al*. (2016) found that health professionals did not acknowledge diverse sexual orientations or gender identity issues, such as the gender-affirming process was unfamiliar to professionals. In several studies, health professionals reported their lack of knowledge and skills to provide care for SGM youth (East and El Rayess, 1998; Sawyer *et al*., 2006; Kitts, 2010; Rose and Friedman, 2013; Knight *et al*., 2014; Mahdi *et al*., 2014; Torres *et al*., 2015; Vance *et al*., 2015; Shires *et al*., 2017). For example, Torres *et al*. (2015) and Shires *et al*. (2017) found that health professionals were not familiar with gender-affirming medical care for transgender youth and did not know how and when to proceed with this.

#### Element 2. False assumptions about SGM youth

Health professionals may have several false assumptions about SGM youth. The most frequently reported assumption in the studies was a heterosexual assumption (East and El Rayess, 1998; Scherzer, 2000; Ginsburg *et al*., 2002; Kitts, 2010; Arbeit *et al*., 2016; Fuzzell *et al*., 2016). Scherzer (2000) and Arbeit *et al*. (2016) identified that heterosexual assumption led to a situation where SGM youth did not receive information that was relevant to them. Assumptions were also linked to certain SGM youths’ identities. In two studies, being gay was linked to having HIV (East and El Rayess, 1998; Ginsburg *et al*., 2002), and Lefkowitz and Mannell (2017) found that some health professionals connected transgender youth with homosexuality, unstable mental health, placement of gender identity within female/male binary, and the presumption that transgender identity was an experimentation or stage of confusion in adolescence.

#### Element 3. The influence of heteronormativity on encounters with SGM youth

Heteronormativity had a negative impact on SGM youth’s experiences of care. Sawyer *et al*. (2006), Rasberry *et al*. (2015), and Arbeit *et al*. (2016) indicated that without health professionals’ open acceptance of SGM people, SGM youth worried about the judgement and intolerance of health professionals, and this negatively affected an SGM youth’s disclosure to health professionals.

The influences of heteronormativity were described not only as negative attitudes and disrespectful behaviour from health professionals but also as mistreatment of SGM youth. Negative attitudes appeared as patronising (Ginsburg *et al*., 2002; Rasberry *et al*., 2015; Fuzzell *et al*., 2016; Snyder *et al*., 2016), stigmatising, or marginalising SGM youth (Scherzer, 2000; Ginsburg *et al*., 2002; Arbeit *et al*., 2016; Fuzzell *et al*., 2016; Rose and Friedman, 2017). Disrespectful behaviour appeared in various ways. Sometimes, health professionals underestimated SGM youths’ identities and abilities to define themselves because of their young age (Scherzer, 2000; Mahdi *et al*., 2014; Arbeit *et al*., 2016; Snyder *et al*., 2016). Some studies reported that when SGM youth disclosed their identity, health professionals reacted to this disclosure with varying, even intense, ways. The reports included professionals being confused (Fuzzell *et al*., 2016), reserved (East and El Rayess, 1998), or unable to interact with the SGM youth (Knight *et al*., 2014; Mahdi *et al*., 2014). In Snyder *et al*.’s (2016) study, SGM youth described that some health professionals ignored their disclosure, suggested the youth changing sexual orientation to heterosexual (Snyder *et al*., 2016), or gave health information based on their heteronormative attitudes. Scherzer (2000) also found the latter reaction in her study. Ginsburg *et al*. (2002), Scherzer (2000), and Arbeit *et al*. (2016) discovered that a SGM youth’s disclosure to a health professional had, in some cases, resulted in an intense reaction from a health professional, such as leaving from the appointment room.

Heteronormativity also influenced the results of SGM youths’ healthcare. False assumptions about SGM youth led to situations that can be described as mistreatment. In seven studies, information and care to SGM youth were given only from a heterosexual aspect, and health professionals did not ask nor discuss the youth’s sexual orientation (Kitts, 2010; Rose and Friedman, 2013; Arbeit *et al*., 2016; Fuzzell *et al*., 2016; Snyder *et al*., 2016; Lefkowitz and Mannell, 2017; Rose and Friedman, 2017). Health professionals in Lefkowitz and Mannell’s (2017) study had taken STD test samples incorrectly from transgender youth because of their confusion about the youth’s sexual organs.

### Diversity-affirming care

#### Element 4. The considerateness of health professionals towards SGM youth

Health professionals can also work beyond the influence of heteronormativity. Their encounters with SGM youth can be described as *considerate*. Considerateness included both mentions from health professionals and SGM youth in the studies. Several studies found that open-mindedness was important for SGM youth (Ginsburg *et al*., 2002; Hoffman *et al*., 2009; Rasberry *et al*., 2015; Arbeit *et al*., 2016; Lefkowitz and Mannell, 2017), as well as equality and respect from health professionals (Ginsburg *et al*., 2002; Hoffman *et al*., 2009; Snyder *et al*., 2016). Both professionals and SGM youth indicated that considerate health professionals can create a comfortable and safe environment for SGM youth (Scherzer, 2000; Ginsburg *et al*., 2002; Arbeit *et al*., 2016; Snyder *et al*., 2016; Lefkowitz and Mannell, 2017). Considerateness came out as health professionals’ genuine care about SGM youth and their health in Torres *et al*. (2015) results. Knight *et al*. (2014) and Lefkowitz and Mannell (2017) found that health professionals were able to identify diversity in sexuality and gender, and that health professionals supported the youth in decision-making and reaching sexual health information. In four studies, health professionals who indicated having knowledge gaps about SGM youth and their health, considered training and education about these topics to be significant (East and El Rayess, 1998; Knight *et al*., 2014; Vance *et al*, 2015; Shires *et al*., 2017).

#### Element 5. Inclusive care of SGM youth

Torres *et al*. (2015), Arbeit *et al*. (2016), and Fuzzell *et al*. (2016) highlighted the importance of acceptance and normalisation of diversity in sexuality and gender when organising inclusive care for SGM youth. In other studies, inclusive care was considered from three aspects: health professionals, information, and healthcare setting.

***Inclusive health professionals*** were described as users of inclusive language (e.g. the neutral *partner* than the assumed heterosexual opposite) (Hoffman *et al*., 2009; Fuzzell *et al*., 2016), who asked which pronouns gender minority youth preferred (Torres *et al*., 2015; Fuzzell *et al*., 2016). Several studies indicated that inclusive health professionals had knowledge about issues related to SGM youths’ health and well-being (Ginsburg *et al*., 2002; Hoffman *et al*., 2009; Rasberry *et al*., 2015; Torres *et al*., 2015; Fuzzell *et al*., 2016; Snyder *et al*., 2016; Rose and Friedman, 2017) and diversity in sexuality and gender (Ginsburg *et al*., 2002; Knight *et al*., 2014; Torres *et al*., 2015; Arbeit *et al*., 2016; Fuzzell *et al*., 2016). Related to interpersonal skills, inclusive health professionals were described as having an ability to meet the youth sensitively (Scherzer, 2000; Hoffman *et al*., 2009; Snyder *et al*., 2016). Some studies stated that health professionals needed an ability to promote open discussion with SGM youth (Rose and Friedman, 2013; Rasberry *et al*., 2015; Arbeit *et al*., 2016; Fuzzell *et al*., 2016; Snyder *et al*., 2016).

***Inclusive information***consisted of topics that were specific to SGM youths’ health, as well as information related to other perspectives in life. Many studies mentioned the sexual health of SGM youth as an important health information topic (Ginsburg *et al*., 2002; Kitts, 2010; Arbeit *et al*., 2016; Snyder *et al*., 2016; Rose and Friedman, 2017), as well as information about sexual orientations (Ginsburg *et al*., 2002; Kitts, 2010; Snyder *et al*., 2016), gender identities (Ginsburg *et al*., 2002), and mental health (Ginsburg *et al*., 2002; Hoffman *et al*., 2009; Snyder *et al*., 2016). Ginsburg *et al*. (2002), Hoffman *et al*. (2009), and Arbeit *et al*. (2016) reported SGM youth were willing to get information about how to talk with parents about their identities, and in the studies of Ginsburg *et al*. (2002) and Arbeit *et al*. (2016), SGM youth were interested about human rights issues related to them.

***Inclusive healthcare settings*** were described in six studies. First, Ginsburg *et al*. (2002), Hoffman *et al*. (2009), Torres *et al*. (2015), and Fuzzell *et al*. (2016) mentioned that an inclusive healthcare setting needed to have a sign of inclusivity for SGM youth, such as stickers (rainbow or pink triangle), informational leaflets and posters, or SGM-oriented magazines. Second, three studies recommended that medical forms should use inclusive language (Torres *et al*., 2015; Fuzzell *et al*., 2016; Snyder *et al*., 2016) and an option for gender minority youth to define their gender identity outside of the female/male binary (Knight *et al*., 2014; Fuzzell *et al*., 2016). Third, SGM youth who participated in the study of Ginsburg *et al*. (2002), Hoffman *et al*. (2009), or Snyder *et al*. (2016), indicated an interest in health clinics that specialised in SGM youth. However, Ginsburg *et al*. (2002) and Hoffman *et al*. (2009) also described how some SGM youth thought these clinics were isolating and labelling from other young people.

## Discussion

This integrative review was the first describing the encounters with SGM youth in healthcare based on the existing research. This review revealed insights about the role of heteronormativity in the encounters between SGM youth and health professionals. It also identified elements of diversity-affirming care. Thus, this review discovered that the diversity of SGM youth is not always recognised, but elements supporting diversity in healthcare also exist. We suggest that further research could study how diversity-affirming care elements could be applied to the healthcare of SGM youth. The review focused on heteronormativity, which is based on feminist research (Jackson, 2006). Future research could focus on another feminist research approach, intersectionality. Intersectionality identifies how different identities such as race, class, gender and sexuality intersect each other, and how social power related to these identities affect to person’s status for example oppressively (Van Herk *et al*., 2011). Intersectionality could raise up new issues in the healthcare of SGM youth, since they can be a diverse group from other aspects besides gender and sexuality (Jackson, 2006).

One of the most commonly reported element of heteronormativity was a heterosexual assumption about SGM youth in healthcare (East and El Rayess, 1998; Scherzer, 2000; Ginsburg *et al*., 2002; Kitts, 2010; Arbeit *et al*., 2016; Fuzzell *et al*., 2016). This issue has been recognised in previous literature (O’Neill and Wakefield, 2017; Brooks *et al*., 2018), and its influences for SGM youth were identified in this review. One example of the influence of the heterosexual assumption in healthcare is lack of information that is relevant to SGM youth. This was reported often—a total of 11 studies in this review. Earlier literature has also found the same issue in the healthcare of SGM youth (Garbers *et al*., 2017; Steinke *et al*., 2017). This aspect reflects how heteronormativity still affects different fields in life including healthcare. SGM youth often have limited protective support resources (Hirsch *et al*., 2010), and if they cannot access relevant information in healthcare, they may look for it elsewhere, such as from online resources (Gay, Lesbian & Straight Education Network *et al*., 2013; Steinke *et al*., 2017). The results of this review showed that SGM youth desired to have access to information related to their health and well-being from open-minded, youth-respecting health professionals (Ginsburg *et al*., 2002; Hoffman *et al*., 2009; Rasberry *et al*., 2015; Arbeit *et al*., 2016; Snyder *et al*., 2016). Thus, SGM youth need interactions with adults about their development, health, and well-being. Online resources can support, enable, or even enhance this interaction.

An integrative review has the potential to describe the complexity of a phenomenon from various perspectives (Whittemore and Knafl, 2005). In this integrative review, over half of the studies were conducted from health professionals’ perspectives (East and El Rayess, 1998; Sawyer *et al*., 2006; Kitts, 2010; Rose and Friedman, 2013; Knight *et al*., 2014; Mahdi *et al*., 2014; Torres *et al*., 2015; Vance *et al*., 2015; Lefkowitz and Mannell, 2017). This shows how SGM youth were often studied from an external view, and the description of the role of heteronormativity might be, at some point, biased in this review. However, the review is the first to focus on a specific aspect in the healthcare of SGM youth, and the results give a new understanding about heteronormativity that evidently affects this youth minority group. Achieving this understanding is significant, when health professionals want to understand diversity in young people, improve their awareness of SGM youth and their challenges in life, and provide care ensuring healthy transitions to adulthood (Moon *et al*., 2002). The transferability of the results may be limited to other healthcare systems and settings besides American, Canadian, or United Kingdom (Polit and Beck, 2010), since healthcare policies and economic factors related to healthcare can vary between countries. However, the results show that more attention is needed regarding how SGM youth are encountered in different healthcare systems globally, and how heteronormativity affects the equality in adolescents’ healthcare. Neither can we forget that even if the recognition of same sex relationships has advanced globally within past years (International Lesbian, Gay, Bisexual, Trans and Intersex Association, 2017; Smalley *et al*., 2018), equality in healthcare for all SGM youth groups has still not achieved; for example, the healthcare of transgender youth is still missing evidence-base (World Medical Association 2015; de Vries *et al*., 2016). Recently, World Health Organization removed transgender identity from the category of ‘mental, behavioural and neurodevelopmental disorders’ to ‘conditions related to sexual health’ in the International Classification of Diseases (ICD11) list (World Health Organization 2018b). This shows an improvement in understanding gender diversity. However, significant work is still needed to ensure that healthcare and medical guidelines take a step to pro-trans direction in practice, and this promotes recognising the needs of transgender youth in particular.

This review showed significant issues related to transgender youth care. First, two studies defined transgender as a sexual orientation (Ginsburg *et al*., 2002; Snyder *et al*., 2016), thus differing from the definition of trans as a gender identity in the literature (Smalley *et al*., 2018). This indicates that the understanding of gender identities may not be congruent in research yet. Second, although Vance *et al*. (2015), Torres *et al*. (2015), Lefkowitz and Mannell (2017), and Shires *et al*. (2017) studied transgender youth healthcare from health professionals’ perspectives, no studies focussed on transgender youths’ perceptions about healthcare. Third, several studies on SGM youths’ perspectives included transgender youth in their study (Ginsburg *et al*., 2002; Hoffman *et al*., 2009; Fuzzell *et al*., 2016; Snyder *et al*., 2016) by grouping them together with sexual minority youth. However, grouping transgender youth and sexual minority youth together may cause prioritising sexuality over gender issues in studies (Steinke *et al*., 2017). Transgender youth have some unique aspects in healthcare such as hormone treatments, gender dysphoria, right to define their gender to their medical files (World Professional Association for Transgender Health, 2011); therefore, the results in this review cannot be generalised to transgender youths’ healthcare. Other gender minority youth were rarely included in the studies, and this showed a significant research gap about gender diversity among young people. There is a need for research focussing on the perspectives of transgender and other gender minority youth in healthcare and how gender norms are affected in healthcare (Steinke *et al*., 2017). Furthermore, the results showed health professionals lacked knowledge about the health issues of transgender youth (Torres *et al*., 2015; Fuzzell *et al*., 2016; Shires *et al*., 2017). More attention in research should consider how health professionals’ knowledge gaps can be diminished.

The review has several practical implications. First, the results show health professionals need education about SGM youth, and the results can be used as a theoretical framework for education about diversity-affirming care. Second, health professionals can use the results in their practice when they want to be inclusive for SGM youth, for example through neutral language and not making assumptions from their patients’ gender and sexuality. Third, when healthcare policies and practices are developed into diversity-affirming, the results give examples of elements for creating inclusive healthcare settings and information for SGM youth.

### Limitations

Some limitations are worth noting in this review. First, the literature search used six databases, but only 18 research articles were found to be eligible for this review. However, the literature search was planned and performed carefully. Two authors did the search systematically by following the same search strategy, and they screened and selected research articles in cooperation. Second, the wide range of research methods used in the studies may affect the analysis and results of this review. This review aimed to describe the role of heteronormativity with a broad approach and not be limited by studies with a specific research method. Furthermore, the quality appraisal of the studies was done by two authors to get an overview of the differences between studies in their quality. Third, the quality appraisal showed that several studies in this review were low in quality, thus we recommend the results are considered with a carefulness. This review was, however, describing heteronormativity for the first time with a broad approach, and the exclusion of studies with low quality was considered incongruent with this. Finally, as one inclusion criterion was English language, the review may have missed eligible studies published in other languages.

### Summary

The results of this review provide a new understanding about the encounters between SGM youth and health professionals in healthcare. This understanding addresses how heteronormativity is related to these encounters and how an open-minded encounter is possible for SGM youth by giving them enough space to be diverse and support for their needs. Further research is needed about the role of heteronormativity in healthcare and the application of diversity-affirming care into healthcare practices. In addition, transgender and other gender minority youths’ voices need to be heard in research. With further research, health professionals may be able to develop their skills when encountering SGM youth without the influence of heteronormativity, give information and support that are relevant to them, and adequately understand how diversity in sexuality and gender is encountered with young patients.
